# Current advances for *in vitro* protein digestibility

**DOI:** 10.3389/fnut.2024.1404538

**Published:** 2024-05-30

**Authors:** Guillermo Santos-Sánchez, Beatriz Miralles, André Brodkorb, Didier Dupont, Lotti Egger, Isidra Recio

**Affiliations:** ^1^Institute of Food Science Research, CIAL (CSIC-UAM, CEI UAM+CSIC), Madrid, Spain; ^2^Teagasc Food Research Centre, Fermoy, Ireland; ^3^INRAE – Institut Agro, STLO, Rennes, France; ^4^Agroscope, Bern, Switzerland

**Keywords:** *in vitro* protein digestibility, protein nutritional quality, *in vitro* DIAAS, simulated gastrointestinal digestion, INFOGEST

## Abstract

Protein is an essential macronutrient in our diet, source of nitrogen and essential amino acids, but the biological utilization of dietary protein depends on its digestibility and the absorption of amino acids and peptides in the gastrointestinal tract. The methods to define the amount and the quality of protein to meet human nutritional needs, such as the Digestible Indispensable Amino Acid Score (DIAAS), require the use of animal models or human studies. These *in vivo* methods are the reference in protein quality evaluation, but they are expensive and long-lasting procedures with significant ethical restrictions. Therefore, the development of rapid, reproducible and *in vitro* digestion methods validated with *in vivo* data is an old demand. This review describes the challenges of the *in vitro* digestion methods in the evaluation of the protein nutritional quality. In addition to the technical difficulties to simulate the complex and adaptable processes of digestion and absorption, these methods are affected by similar limitations as the *in vivo* procedures, i.e., analytical techniques to accurately determine bioavailable amino acids and the contribution of the endogenous nitrogen. The *in vitro* methods used for the evaluation of protein digestibility, with special attention on those showing comparative data, are revised, emphasizing their pros and cons. The internationally harmonized digestion protocol proposed by the INFOGEST network is being adapted to evaluate protein and amino acid digestibility. The inter-laboratory reproducibility of this protocol was demonstrated for dairy products. The *in vivo/in vitro* comparability results obtained to date with this protocol for several plant and animal sources are promising, but it requires an extensive validation with a wider range of foods and substrates with known *in vivo* digestibility. These *in vitro* methods will probably not be applicable to all foods, and therefore, it is important to identify their limitations, not to elude their use, but to apply them within the limits, by using the appropriate standards and references, and always as a complementary tool to *in vivo* tests to reduce their number.

## 1 Introduction

Protein is an essential macronutrient in our diet, source of nitrogen and essential amino acids. In human nutrition, the term protein nutritional quality refers to the ability of a protein to meet human requirements in essential amino acids and fulfill the physiological needs (1). The biological utilization of dietary proteins depends on their digestibility and the absorption of amino acids and di- and tri- peptides in the gastrointestinal tract. Protein digestibility is linked to its unique amino acid composition, which, in turn, determines the folding state of the protein. For instance, the gastric survival of some globular proteins, such as milk β-lactoglobulin is well known (2), as well as the intestinal resistance of proline-rich protein domains due to the limited intestinal cleavage of the amide bond of proline residues (3). Post-translational modifications, especially glycosylation and phosphorylation, also confer additional gastrointestinal resistance to the protein, as occurs for casein phosphorylated regions that have been found at different sections of the gastrointestinal tract (4, 5). In addition, proteins are often included in supramolecular structures, such as, protein bodies, micelles, fibers, or entrapped in cellular structures surrounded by non-digestible polysaccharides that limit the access of gastrointestinal enzymes (6, 7). Additionally, food products are commonly subjected to different technological processes to improve sensory properties, ensure safety or extend shelf-life, and these processes can also affect protein digestibility. While soft heat treatments denature globular proteins and inactivate anti-nutritional factors which increase digestibility, more severe treatments lead to protein aggregation, cross-linkages, or non-enzymatic browning, decreasing digestibility (8).

Given the complexity of the digestion and absorption processes and the importance to accurately define the amount and the quality of protein required to meet human nutritional needs, protein quality evaluation is being subject to numerous studies and updates. As a result of the FAO Expert Consultation on Protein Quality Evaluation in Human Nutrition held in 2011, a new protein quality index, the Digestible Indispensable Amino Acid Score (DIAAS) was proposed to replace the Protein Digestibility Corrected Amino Acid Score (PDCAAS) (9). DIAAS reflects the balance of amino acid digestibility determined at the terminal ileum, and describes protein quality better than PDCAAS (10). For the calculation of DIAAS, protein digestibility is based on the true ileal digestibility of each amino acid, preferably determined in humans, but if this is not possible, in growing pigs or in growing rats, in that order. In this report, the importance of treating each indispensable amino acid as an individual nutrient was also highlighted, and therefore, the digestibility is calculated as the oro-ileal disappearance of each amino acid. A dataset is being built based on the true or standardized ileal amino acid digestibility of a wide range of foods and ingredients. However, all these indexes, PDCAAS and DIAAS, and other previously used methods like the protein efficiency ratio (PER), include the use of animal models, or human studies. Consequently, dietary protein is the sole food macronutrient that requires animal or human testing for regulatory purposes.

These *in vivo* methods, although are the “gold standard” in protein quality evaluation, have important drawbacks. Animal trials are expensive and long-lasting methods with ethical restrictions. In addition to the policies on experimental animals that lead to follow the principle of the 3Rs (replacement, reduction and refinement), the social demand to reduce the number of animals for experimental purposes is currently growing, as well as, the demand for animal-free food, motivated by environmental and animal welfare reasons. In addition, because protein digestibility is affected by the food matrix and food composition and the technological treatment or the cooking conditions applied, the number of trials to be run exponentially increases, and makes the use of animal or human tests unfeasible.

Therefore, the development of rapid, reproducible and *in vitro* digestion methods that allow the estimation of the protein nutritional quality is an old demand. Despite the huge efforts done, especially in the field of animal nutrition [reviewed by Moughan (11)], these methods have not been sufficiently validated with appropriate *in vivo* data, i.e., ileal and not fecal protein digestibility, to reach sufficient confidence. The aim of this review is to present the actual status of the available *in vitro* methods to calculate protein digestibility with a view on past developments and special focus on the *in vivo/in vitro* comparability. The scope, uses and limitations of these *in vitro* procedures will be discussed, as well as, the work needed for the future application of these methods in routine protein quality evaluation.

## 2 Challenges of the *in vitro* methods

### 2.1 Simulate the *in vivo* digestion: a difficult task

Over the last 40 years, there has been interest in simulating human digestion *in vitro*, and specifically protein digestibility, since this knowledge is crucial in different areas, going from the nutritional assessment of novel foods and ingredients to the evaluation of protein allergenicity. However, human digestion and absorption are complex, multistage and adaptable processes in which several factors are involved (12). Thus, *in vitro* simulation of the digestion and absorption is a technically difficult, if not impossible, task. In this sense, although there are conditions that can be reproduced *in vitro*, with more or less success, such as, gastrointestinal enzymes, coenzymes and cofactors, pH, or temperature, there are other variables, such as, mechanical forces, regulation by gastrointestinal hormones, action of the intestinal microbiota or the participation of other organs, that are difficult to reproduce (13, 14). Furthermore, several studies have shown that the digestive capacity is adaptable, and for instance, the enzyme release at different levels of the gastrointestinal tract is regulated by the amount of ingested food (15, 16). This aspect, together with the ability of the products of digestion to modulate the intestinal function, adds extra complexity to the digestive process (17, 18). Although gut microbiota plays a critical role in digestion, its effects on protein quality evaluation could be overlooked in an *in vitro* approximation, since the goal of these methods is to simulate gastrointestinal digestion up to the ileum.

### 2.2 Simulate absorption and analysis techniques

Another important challenge of the *in vitro* methods to evaluate protein digestibility is the definition of the digestible-, bioavailable- or absorbable-fraction. Because the calculation of *in vivo* protein digestibility is based on the difference between the amount of each amino acid ingested and that non-absorbed, many *in vitro* methods have tried to reproduce or approximate these digestible and non-digestible fractions through dialysis, filtration or protein precipitation (14). However, other methods have estimated protein digestibility in the whole digest, like in the pH-drop or pH-stat methods, as will be described later on. However, as it will be commented, these methods based on pH measurement or monitoring were found to be susceptible to the buffering capacity of components of some food materials (19, 20).

In order to improve the evaluation of protein and amino acid digestibility, the resulting digestible and non-digestible fractions can be analyzed by using the same analytical approaches as the ones used in the *in vivo* assays, i.e., total nitrogen by Kjeldahl or Dumas, and determination of total amino acids by gas chromatography (GC) or HPLC. The different methodologies used for protein quantification can generate discrepancies in the data. The Kjeldahl method is considered the standard method for the estimation of nitrogen in food, because of its universality and good reproducibility in liquid and solid samples. Kjeldahl is a chemical method that determines the nitrogen concentration released during digestion with strong acids (21). Dumas is a high temperature combustion method, and the elemental nitrogen is detected by a thermal conductivity detector. However, because not all of the food nitrogen comes from proteins, these two methods do not give a measure of the true protein. Results obtained with Dumas are usually a little bit higher than those with Kjeldahl due to the detection of nitrogen compounds like nitrates, nitrites and heterocyclic compounds that are not completely quantified by Kjeldahl. In this sense, both methods might need determination of the non-protein nitrogen fraction depending on the food evaluated, and more importantly, an accurate nitrogen to protein conversion factor (NPCF) to convert the nitrogen values into protein. For many foods and ingredients, an overestimation of protein content can result from the use of the standard nitrogen correction factor 6.25 (22), and this will be translated into an underestimated protein digestibility value.

In both, the *in vivo* and *in vitro* assays, an acidic hydrolysis with 6 N HCl at 110°C has to be performed for 18–24 h prior to total amino acid determination by GC or HPLC. The effect of this acidic hydrolysis on amino acid analysis was questioned by Darragh and Moughan (23), who showed that the hydrolysis process can generate an erroneous estimate of the amino acid composition. This is due to the presence of amino acids that require times greater than 24 h to cleave the peptide bond (isoleucine, leucine, and valine) and others, considered labile amino acids, which can be partially destroyed before measurement (serine and threonine). In this context, proteins produce different rates of release and loss of amino acids, depending on their amino acid composition, causing an inaccurate quantification. Furthermore, the effect of acid hydrolysis on the chemical integrity of amino acids has been a topic of great scientific interest for years. It is well known that during thermal processing and storage, some amino acids such as methionine, cysteine, threonine, and tryptophan become unavailable, decreasing their bioavailability between 1 and 10%, as in the case of histidine (24). This aspect is especially notable for lysine amino acids, which are easily damaged during the food processing. The ɛ-amino group of lysine can react with many compounds such as reducing sugar, vitamins, fats, polyphenols, generating reactions that produce isopeptides and causing a degradation of lysine. Of these reactions, the most important occurs when, during thermal processing, the amino group reacts with the reducing sugar forming early or late Maillard compounds, which generates a decrease in the availability of lysine. When the Maillard reaction is advanced, the lysine is completely destroyed and cannot be recovered. However, during the early stages of the Maillard reaction, a portion of the structurally altered lysine (Amadori products) is partially hydrolysed in the presence of strong acids that reverse to lysine. However, such reversion does not occur during gastrointestinal digestion (25, 26). This fact leads to an overestimation of lysine quantification in processed foods caused by the acid hydrolysis step that takes place during conventional amino acid analysis. For this reason, the ultimate measure of available lysine is considered the absorbed reactive lysine and new methods, such as the isotope method or the oxidation of an indicator amino acid, have been described (27).

Other methods widely used to measure protein hydrolysis degree are those based on the reaction of primary amino groups, such as the trinitrobenzenesulfonic acid (TNBS) or the o-phthaldialdehyde (OPA) procedure. However, the precision of these methods may depend on the method and the protein substrate being hydrolysed. For instance, several studies have shown that OPA and cysteine react weakly due to the sulfhydryl group of cysteine, generating an unstable product (28, 29). This aspect makes the OPA method unsuitable for quantifying the degree of hydrolysis in cysteine-rich substrates. Furthermore, TNBS or OPA do not react with secondary amino acids such as proline or hydroxyproline (30).

### 2.3 Enzymes and blank of enzymes

One of the critical points in the *in vitro* digestion protocols is the selection of the enzymes and conditions (pH, digestion times, and salt concentration) to mimic physiological digestion. To study protein digestibility, proteases of porcine origin that are commercially available, have been widely used, specifically, porcine pepsin for the gastric phase and porcine pancreatic extracts containing proteolytic, lipolytic and amylolytic activities or individual proteolytic enzymes (31). The physiological protease concentrations at different segments of the gastrointestinal tract have been revised to fix conditions in some *in vitro* protocols (32, 33). In addition, other methods included peptidases of bacterial origin to simulate the carboxy- and amino-peptidase activity of intestinal brush border enzymes (34). It is true that when the food contains a high fat or starch content, an insufficient digestibility of these macronutrients can affect protein digestibility, and amylolytic and lipolytic enzymes are less accessible or are available at high prices. Starch digestion starts in the oral phase and, and although it is inactivated by the low pH in the stomach, some activity may persist within the food bolus. However, oral amylase is not included in most protocols due to its high price. Similarly, lipid digestion starts in the stomach by the action of gastric lipase that reaches activities of ca 120 U/mL in gastric fluid (35). Due to the limited accessibility of human gastric lipase, and taking into account the triglycerol stereospecificity and pH stability, dog or rabbit gastric lipases have been proposed as closest substitutes (36, 37), however gastric lipase is not used in most of the *in vitro* methods to evaluate protein nutritional quality. Bile salts have also been reported to improve the protein hydrolysis by the action of pancreatic proteases (38), however, not all *in vitro* protocols include conjugated bile acids in the intestinal phase. More importantly, as detailed later on (Section 4), the key to ensure batch-to-batch and inter-laboratory reproducibility is the standardization of enzymatic activity. Most of the *in vitro* digestion protocols add a given amount of enzyme or fix an enzyme/substrate ratio (E/S) on weight basis. This adds an important source of variability since the enzymatic activity of commercial enzymes varies enormously from batch to batch and during prolonged or inappropriate storage.

During the simulated gastrointestinal digestion, the use of enzymes, pancreatic extracts or mucins adds a significant amount of protein that depends on the E/S. In a similar way as occurs in *in vivo* trials, the amount of “added nitrogen” needs to be subtracted in the final calculations of protein and amino acid digestibility. In those methods that separate the digestible and non-digestible protein fractions, this deduction should be done in one or in both fractions. Due to the high degree of autolysis of the digestive enzymes, in particular in absence or in low dietary protein concentrations (39), the use of water or simulated physiological fluids as blank will affect the calculation, underestimating digestibility.

### 2.4 Other factors that affect protein digestibility

The presence of antinutritional factors (ANF), i.e., lectins, saponins, polyphenols, or trypsin inhibitors, or the fiber content can also influence *in vivo* protein digestibility. ANF could compromise the protein digestibility by inhibiting the accessibility of the digestive enzymes to the protein or inhibiting enzyme activity (40). Trypsin inhibitors, found in field pea, peanut, wheat, lupin, and soybean, have been demonstrated to be capable of reducing the ability to bind the active site of the enzyme, while lectins may interfere with the digestion and absorption of nutrients (41). Furthermore, polyphenols have been shown to generate a complex with the digestive enzymes, inactivating them and therefore reducing the digestibility of proteins (13, 42). Moreover, fiber consumption has shown several effects on the gastrointestinal tract, from reducing enzymatic activity in the lumen and transit time to protecting the enzymes against degradation or stimulating the microbiome activity in the digestive tract. However, the effect of these compounds in the *in vitro* assays is still unknown and will depend on the conditions, especially the E/S used, which makes it difficult to simulate *in vivo* digestion with *in vitro* assays (43).

## 3 Historical overview

During the last decades, different *in vitro* digestion methods have been developed to evaluate protein digestibility, and thus, nutritional quality of foods and feed. The number of articles dealing with this subject is enormous with more than 5,000 publications from 1990 to date. Therefore, this historical overview will be limited to dietary proteins, and with a special emphasis on those works showing *in vivo*/*in vitro* comparative data. Most of these methods are based on enzymatic hydrolysis, performed in a one- or two-step process, by using a single or a combination of enzymes, often being gastrointestinal proteases from porcine origin. Hydrolysis conditions, pH and temperature, are generally fixed at the maximum for each enzyme with pepsin hydrolysis carried out at acidic pH (around pH 2) and pancreatic enzymes used near neutrality. Some methods to evaluate *in vitro* digestibility of dry matter in feedstuffs proposed to account for microbial degradation at the large intestine by adding a multienzyme step containing a wide range of carbohydrases including cellulase, hemicellulose, arabinose, xylanase and others (44, 45). Differences between approaches are given by the E/S and especially by the method employed to determine protein digestibility. Some methods are based on a measurement of pH (pH drop or pH stat) while others are based on the separation of a digestible or absorbable fraction by various procedures going from ultrafiltration or dialysis to the use of protein precipitating agents. Therefore, in this section, *in vitro* methods are classified by the principle used to evaluate protein digestibility. Table 1 collects different *in vitro* methods used to assess protein and amino acid digestibility in comparison to *in vivo* data where the limitations have been specified.

**Table 1:** *In vitro* methods used to assess protein and amino acid digestibility in comparison to *in vivo* data.

Food substrate	*In vitro method*	*In vivo* model	Outcome	Limitations	Reference
**pH drop**					
23 human diets (plant and milk proteins, and food products)	3-enzyme^*^ method	Apparent fecal protein digestibility, PER (Rats)	High correlation (r = 0.90) between pH-drop and *in vivo* apparent digestibility	Affected by buffering capacity of food	(19)
61 samples of food and feed [plant, combination (plant–animal) and animal proteins]	3-enzyme^*^ and 4-enzyme^**^ methods	Standardized fecal protein digestibility (Rats)	High correlation (r = 0.89–0.90), for plant proteins and for combination proteins	Animal proteins were underestimated Correction for buffer capacity of foods is needed 3-enzyme method affected by tannins	(46)
60 vegetable proteins (cereal grains, leguminous seeds, oilseeds, and by-products)	4-enzyme^**^ method	Apparent fecal protein digestibility (Rats)	Overall r = 0.838, but differences between food groups	Distinct equations for different groups of samples	(47)
20 meat and bone meal samples	4-enzyme^**^ method	Standardized ileal protein digestibility (Rats)	r > 0.75	Affected by the buffering capacity of the ash content	(20)
Soy protein concentrate	pH-drop vs. SDS-PAGE 4-enzyme^**^ method *vs* Pepsin 2 h + pancreatin 6 h	Apparent fecal protein digestibility (Rats)	pH-drop correlation r = 0.95	Discrepancies with SDS-PAGE due to protein aggregates	(48)
30 protein samples (animal, plant and combinations of plant–animal proteins)	pH-drop and pH-stat 3-enzyme^*^ and 4-enzyme^**^ methods	Standardized fecal protein digestibility (Rats)	r = 0.78 and 0.56 for pH-drop, depending of the enzyme method r > 0.90 for pH-stat	Pre-digestion with pepsin is suggested for samples containing proteinase inhibitors	(49)
**pH stat**					
Maize Whole sorghum Pearled sorghum	3 different methods: Pronase Pepsin 3-enzyme^*^ method	Apparent fecal protein digestibility (Rats)	pH-stat procedure correlated better (r = 0.95) than systems containing pronase and pepsin *in vitro*	Multienzyme: highest correlation vs. *in vivo* Pepsin: poor correlation vs. *in vivo*	(50)
17 foods (animal and plant proteins)	Pepsin +4-enzyme^**^ method	Standardized fecal protein digestibility (Rats)	*R*^2^ = 0.61 all foods *R*^2^ = 0.66 without beans and chickpeas	Low correlation values Poor correlation for beans and chickpeas (fecal digestibility)	(16)
10 salmonid diets	3-enzyme^*^ and 4-enzyme^**^ methods	*In vivo* digestibility by chromic oxide method (Fish)	*R*^2^ = 0.82 and 0.64 depend on the pH-stat method used	Overestimation or underestimation depending on diet and method used.	(51)
7 feed ingredients (menhaden, Atlantic menhaden, anchovy, white fish, tuna waste, soybean protein, and langostilla meals)	Shrimp hepatopancreas enzymes or a multienzyme solution^**^	Apparent fecal protein digestibility (White shrimp)	*R*^2^ ≈ 0.71 or 0.77 depending on the enzymes used	Low correlation values Additional *in vivo* data are needed	(52)
A veal protein hydrolysate vs. gelatin vs. caseinate	3-enzyme^*^ method	PER and standardized fecal protein digestibility (Rats)	Linear relationship between *in vivo* digestibility and pH-stat method (*R*^2^ = 0.99)	One substrate The use of published regression equations is unreliable	(53)
Soybean and retoasted soybean meals Rapeseed and retoasted rapeseed meals	2 *in vitro* methods: 3-enzyme^*^ pH-stat Pepsin + pancreatin	Standardized ileal protein digestibility (Growing pigs)	Both *in vitro* methods correlated with *in vivo* digestibility (r = 0.95; r = 0.91)	2 plant substrates with 2 treatments	(54)
**Precipitation methods**					
4 experimental diets (corn, barley, oats, soybean, corn gluten and wheat bran)	1% TCA Pepsin 6 h pH 1 + pancreatin + amylase 1 h pH 6.8	Ileal digestibility (Broilers)	Correlation with digestibility of crude protein r = 0.93 when diets ground to 0.4 mm	Better results with highly digestible diets than diets of low digestibility.	(55)
7 plant feedstuffs and 16 diets	2% SSA Pepsin 6 h pH 2 + pancreatin 18 h pH 6.8	Apparent fecal digestibility (Growing pigs)	Linear regression with crude protein digestibility but *in vitro* higher than *in vivo* values r = 0.99 for feedstuffs r = 0.95 for diets (r = 0.8 for unextracted diets)	Fat extracted feeds and diets Only N contents	(56)
17 feedstuffs (15 plant-based meals *vs* meat and bone meal *vs* dairy) and 48 feed mixtures	% SSA Pepsin 6 h pH 2 + pancreatin 18 h pH 6.8	Apparent ileal digestibility (Growing pigs)	Linear relationship *R*^2^ = 0.61 all feedstuffs *R*^2^ = 0.92 excl. Meat and bone meal and barley hull Validation with 48 feeds (*R*^2^ = 0.57) *In vitro* AA digestibility (9 products)	*In vitro* protein digestibility > apparent ileal digestibility Relationship generally higher for essential AA, and lower for non-essential AA, than for protein	(57)
28 samples of dry extruded dog foods	2% SSA vs. pH-drop-3 enzyme^*^ method *vs* Near infrared spectroscopy	Apparent fecal protein digestibility (Dogs)	Correlation with *in vivo* crude protein digestibility: Protein precipitation r = 0.81; pH-drop r = 0.78. Near infrared spectroscopy *R*^2^ cv. = 0.53	The ash content affects the accuracy of the pH-drop-method	(58)
**Dialysis cell**					
Protein diets including beef, casein, rapeseed, soybean and gluten	Dialysis cell-1 kDa Pepsin 0.5 h pH 2 + pancreatin 6 h pH 6.8 *vs* pH stat^*^	Portal and aortic blood (Rats)	r = 0.92 for plant sources r = 0.70 for animal sources	Variation between protein groups Poor correlation for animal sources	(59)
Heated rapeseed meal, soybean, lupine proteins vs. sodium caseinate vs. gelatin	Dialysis cell-12 kDa Pepsin 4 h pH 2 + trypsin 24 h vs. pH-stat	Fecal digestibility and PER (Rats)	r = 0.88 (true digestibility vs. dialysis cell) r = 0.81 (true digestibility vs. pH-stat)	Comparison with fecal digestibility Only 1 animal protein (gelatin)	(60)
3 feedstuffs: Fish meal, rapeseed meal, cottonseed meal	Dialysis cell-12 kDa Pepsin 4 h pH 2 + trypsin 24 h	Apparent ileal digestibility (Black pig barrows)	Linear regression 0.96 < r < 0.99 Significant linear relationships between ileal apparent digestibilities for crude protein, total AA and 16 individual AA	Comparison with apparent digestibility	(61)
17 grain legumes (faba beans, field pea, lupin)	Dialysis cell-1 kDa Pepsin 0.5 h pH 2 + pancreatin 6 h pH 6.8	Standardized ileal digestibility (Growing pigs)	*In vitro* digestibility higher than *in vivo* *R*^2^ = 0.73 for Lys *R*^2^ = 0.91 for Cys and Trp	ANF content depress nutrient digestibility *in vivo*	(62)
**Dynamic systems**					
Standard corn-based diet with coarse ground corn, beet, wheat bran, beet pulp	TIM^®^ Dialysis fluids = absorbed Pepsin+lipase+pancreatin	Standardized ileal digestibility (Growing pigs)	Including all diets: non-significant correlation Excluding corn diet: *R*^2^ = 0.99	Starch digestibility was underestimated compared with *in vivo* Digestibility dramatically reduced in the TIM by fibrous ingredients; volume limitation for high-fiber diets.	(63)
Dairy infant formula *vs* 50% pea proteins *vs* 50% faba bean proteins	DIGDI^®^ SEC < 10Ka N corrected for free AA and secretions *N* = absorbed Pepsin+pancreatin	Digestion (Piglets)	PDCAAS-like score and apparent digestibility comparable with literature	System validated for dairy infant formulas	(64)

AA, amino acids; N, nitrogen; RT, room temperature; SSA, sulphosalicylic acid; TCA, trichloroacetic acid; PDCAAS, protein digestibility corrected amino acid score; PER, protein efficiency ratio; SDS-PAGE, sodium dodecyl sulfate polyacrylamide gel electrophoresis. ^*^, three-enzyme method (trypsin, chymotrypsin, and peptidase); ^**^, four-enzyme method (trypsin, chymotrypsin, aminopeptidase and protease from Streptomyces griseus). r, correlation coefficient; *R*^2^, determination coefficient.

### 3.1 Methods based on pH measurement

During protein hydrolysis, release of protons and amino acids from the cleaved peptide bonds results in changes in pH. These methods lie on the correlation between the rate of hydrolysis degree and protein digestibility. Hsu et al. developed a multi-enzyme method (three-enzyme method, trypsin + chymotrypsin + peptidase) for the evaluation of protein digestibility. Specifically, they showed that the pH-drop after 10 min of digestion with the three-enzyme solution of 23 human diets, mainly vegetables and dairy foods, was highly correlated (r = 0.90) with *in vivo* PER values in rats. However, substances with high buffering capacities could affect the results. Despite this, the pH-drop methods was able to predict the apparent digestibility of proteins. In addition, the trypsin inhibitory activities and the effect of heat processing on digestion could be detected (19). Two years later, Satterlee et al. slightly modified the Hsu et al. protocol by including an extra 10 min of digestion with a *Streptomyces griseus* protease (four-enzyme method). Numerous authors have evaluated the *in vitro* digestion process of several food sources through the pH-drop method. In 1981, Petersen and Eggum studied the applicability of the three-enzyme (19) and four- enzyme combinations (65) on 61 samples of food and feed. Their results demonstrated a high correlation (r = 0.89–0.90) between the pH-drop results and fecal protein digestibility in rats, especially for plant proteins and for mixtures of plant and animal proteins. However, the predicted *in vitro* digestibility value for animal proteins significantly differed from the *in vivo* results (46). In 1983 the same researchers evaluated the *in vitro* protein digestibility of 18 protein sources using the three-enzyme method of Hsu et al. and the four-enzyme method of Satterlee et al. The results showed a greater *in vitro-in vivo* correlation for the three-enzyme method (r = 0.78) than for the four-enzyme method (r = 0.56). Despite the good correlations obtained, the estimations were significantly affected by the different buffering capacities of some food substances, which was considered a major drawback of the pH-drop method (49). This aspect was further demonstrated by Moughan et al. who compared the *in vitro* digestibility of 20 meat and bone meal samples by the pH-drop method with the values of true ileal digestibility in rats. It was concluded that pH estimation methods may be influenced or affected by the strong buffering capacity of the ash content, mainly mineral content, of food (20). For this reason, it was recommended to determine the pH-drop after a dialysis treatment to eliminate salts with buffering capacities (20). Other authors such as Kim et al. and Wolzak et al. showed *in vitro-in vivo* correlation values of r = 0.95 and r = 0.421 for soy protein concentrate, and 33 vegetable proteins, respectively (47, 48). However, the difference in the response for different types of food proteins made it necessary to use different regression equations to obtain realistic estimates of digestibility. This task presents a major challenge due to the complexity involved in categorizing foods in each class of food.

Pedersen and Eggum revised the pH-drop method and modified it slightly in order to avoid the effect of substances present in the protein that could influence the drop in pH. In the pH-stat procedure, the pH is kept constant at pH 8 by automatic titration (0.10 M-NaOH titrant) during the incubation with enzymes. At the end of the incubation period, the amount of alkali added is recorded and the value is used as an indirect measure of protein digestibility (43, 46). Using pH-stat, Pedersen & Eggum showed an improvement in the prediction of protein digestibility of 30 samples, compared to the pH-drop. A high correlation coefficient (r > 90) with fecal digestibility in rats was obtained in pH-stat method, improving the one obtained by the pH-drop method (0.56–0.78). However, the digestibility of some foods, such as egg powder, was underestimated, because of the content of trypsin and chymotrypsin inhibitors of egg. A pretreatment with alkali to improve the correlation coefficients was then recommended (49). The pH-stat method has been widely used to evaluate the digestibility of different protein sources. Eggum et al. showed a good agreement *in vitro* vs. *in vivo* (measured by fecal protein digestibility in rats) in 17 foods, with the exception of two legumes, beans and chickpeas. The authors discussed that the discrepancies obtained for these foods, suggesting that it could be due to the high bacterial growth with the consumption of certain legumes in the diet, which caused an increase in the excretion of nitrogen in the feces. Excluding these legumes the obtained *in vitro* and *in vivo* digestibility percentages were similar (86.3–100.0% *in vitro* and 73.1–96.8% *in vivo*), although the correlation coefficient was only acceptable (*R*^2^ = 0.66) (16). Better correlation coefficients were obtained by comparing the *in vitro* protein digestibility of maize, whole sorghum and pearled sorghum maize (r = 0.95) and 7 specific foods (*R*^2^ ≈ 0.75) with their *in vivo* apparent fecal protein digestibility (50, 52). In the same way, good and significant correlations (*R*^2^ = 0.82 and 0.64) were obtained when the protein digestibility of 10 salmonid diets were estimated by two the pH-stat *in vitro* assay methods and compared with *in vivo* digestibility in fish (51). Linder et al. measured the protein digestibility of an industrial veal protein hydrolysate, used as a gelatin-replacing ingredient for human consumption. The results showed a high correlation between fecal protein digestibility measured in rats and the pH-stat method (*R*^2^ = 0.99), although already published regression equations were used (53). Recently, high correlation coefficients were obtained between *in vitro* protein digestibility of processed soybean meal and rapeseed meal through the pH-stat method and the results obtained from standardized ileal digestibility in growing pigs (r = 0.95) (54).

In summary, the methods based on pH measurement were shown to be suitable for predicting digestibility in many foods, with high correlations in plant substrates. The method was found to be highly reproducible across 6 laboratories that estimated protein digestibility of 17 protein sources by using the 3-enzyme method in a pH-stat (66). However, by using these methods, the results of the entire complex digestion process were evaluated based on a mere measurement of pH or pH-change. In other words, the crucial information on protein digestion that could be extracted from the use of gastrointestinal enzymes was neglected. In addition, the significant differences found for animal proteins, the use of different correlation curves for different samples, and the fact that certain physical and chemical characteristics, such as calcium content or buffering capacity, may prevent an accurate estimation of digestibility, and are important drawbacks for the use of these methods.

### 3.2 Methods based on protein precipitation

In the methods described in this section, enzyme incubations are followed by measurements of the insolubilized material collected after filtration, although in some cases measurements on the filtrate, or alternatively on one of the separated fractions after centrifugation are conducted. Digestibility is then related to *in vitro* solubility or the definition of an absorbable or bioaccessible fraction and a residue or non-absorbable fraction.

Early methods included one-step incubations giving lower digestible protein values than those obtained *in vivo*, and were rapidly replaced by two-step digestion. In the pepsin-jejunal fluid, a two-step incubation with pepsin digestion for 4 h followed by a further 4 h digestion with pig jejunal fluid was used (67). *In vitro* digestibility of protein was calculated by the determination of dry matter and crude protein on the residue after centrifugation for 10 min at 1,250 × g at 5° C, on the basis of the original protein content of the diet. A two-stage incubation with pepsin for 6 h at pH 2 followed by an incubation with pancreatin at pH 6.8 for 18 h in borate buffer was further developed (68) thus providing an animal-independent method. To calculate the digestibility, 1% TCA final concentration was used, followed by centrifugation for 1 h at 2,000 × *g*. This method was applied for routine analysis in quality control of feeds and feed ingredients for poultry. By reducing the particle size of the test material, passing through a 0.4 mm sieve, the accuracy of predicting *in vivo* digestibility was increased for all the tested diets, that included corn, barley, oats, soybean, corn gluten and wheat bran as protein sources. The correlations between ileal digestibility in broilers and *in vitro* estimates were high (r = 0.93 for crude protein) (55). A modification of this method was presented by Babinszky et al., where pepsin incubation was performed at pH 1 on fat-extracted feed samples, and the residue after pancreatin + amylase incubation and 1% TCA precipitation was centrifuged at 3,500 × g for 15 min, after decanting over a nylon cloth (particle size 40 μm). This method found an improved correlation with fecal digestible protein in pigs by reaching regression values for feedstuff and diets of 0.99 and 0.95, respectively (56). The additional determination of nitrogen content on the filtrate gave a similar correlation but was abandoned as it was considered to be too laborious.

With the aim to recover solubilized but not fully degraded proteins, precipitation with sulphosalicylic acid was introduced, while undigested materials were submitted to the standardized filtration equipment for measuring dietary fiber (43). Magnetic stirring was included during the enzymatic incubations in order to assure effective starch degradation. By the use of this method, prediction of individual amino acids in eight common feedstuffs showed that the *in vitro* digestibility of the individual amino acids was close to the *in vitro* digestibility of nitrogen. Hervera et al. (58) adapted the last *in vitro* method for estimation of digestible energy of dog foods and compared it with the pH-drop methodology. The results showed a correlation of r = 0.78 between the pH-drop with the three-enzyme method and the apparent fecal digestibility in dogs but higher accuracy, r = 0.81, was shown with the *in vitro* method using precipitation with sulphosalicylic acid (58). Wada and Lönnerdal determined digestibility in infant formulas by using total and non-protein nitrogen (NPN), i.e., soluble fraction in 12% final TCA concentration (69). They investigated the effect of industrial processing with *in vivo* digestibility using a suckling rat pup model in terms of chemical modifications and endurance of intact α-lactalbumin and β-lactoglobulin, but no direct *in vivo-in vitro* comparison was shown.

The role of nitrogen added in the form of enzymes was considered in further developments. When the *in vitro* digestibility of protein was calculated from the difference between nitrogen in the sample and the undigested residue after correction for nitrogen in the blank, it was shown that the resulting amino acid composition of the blank-derived protein was very close to reported values in the literature based on direct measurements of endogenous protein *in vivo*. Apparent ileal digestibility of individual amino acids was predicted in a similar way as for protein. The relationship was generally higher for essential amino acids, and generally lower for non-essential amino acids, than for protein (57). This procedure used precipitation with sulphosalicylic acid (2% final concentration) for 30 min at room temperature followed by rinsing with 1% sulphosalicylic acid of the filtered residues. A close relationship was found for the 17 single feedstuffs but meat and bone meal, and barley hull had to be excluded. The above conditions have been widely used to compare protein digestibility of different products, mainly using sulphosalicylic acid (62, 70) or TCA (71, 72) as precipitating agent.

In summary, the *in vitro* methods based on precipitation of a non-digestible fraction by using different agents such as TCA or sulphosalicylic acid have demonstrated good comparability with ileal digestibility in broilers and in pigs. Precipitation with sulphosalicylic acid after a 3-enzyme digestion protocol has shown higher accuracy than pH-drop when compared with dog fecal digestibility. The main advantage of these methods is the reproducibility of the precipitation step for the definition of a digestible and non-digestible fraction. However, the digestion conditions used by different authors would still require additional optimization and harmonization.

### 3.3 Methods using ultrafiltration or dialysis

These methods are based on the continuous removal of low-molecular-weight products from digested material by ultrafiltration or dialysis to prevent enzyme inhibition by end products.

A two step-digestion method in which the intestinal digestion products (free amino acids and low molecular weight peptides) were removed through a dialysis membrane was proposed in order to reduce enzyme inhibition by hydrolysis products (73). After a 30 min digestion step with pepsin enzyme: substrate of 1:250 (pepsin activity 3,152 units/mg protein), intestinal digestion took place with pancreatin for 6 h, at an E/S of 1:25, in a dialysis cell of a 1,000 Da molecular weight cut-off, for the continuous elimination of digested products with 10 mM sodium phosphate buffer, pH 7.5, as circulating dialysis buffer. The essential amino acids released during the intestinal phase from beef, casein, rapeseed, soybean and gluten correlated with plasma levels found in portal and aortic blood in rats fed with the same substrates (59). A good correlation was found for plant sources (r = 0.92–0.93), although lower values were reported for beef or casein (r = 0.70). This protocol was also applied to protein mixtures (74) and to 19 selected foods, showing differences between *in vitro*-*in vivo* amino acid digestibility depending on the protein source. These variations were not related to the amino acid concentration in the protein and it was proposed that the amino acid sequence as the factor leading overall protein and amino acid digestibility (75). The *in vitro* protein digestibility by use of a dialysis cell method and pH stat was compared with the *in vivo* PER and true digestibility of heated rapeseed meal, soybean and lupine proteins (60). *In vivo,* PDCAAS correlated with pH stat and dialysis cell values with r = 0.92 and 0.98, respectively, although PER was poorly correlated with the *in vitro* protein digestibility. Similar strategies but using dialysis tubes in the intestinal phase have been used to predict ileal protein digestibility of pig feedstuffs (61), obtaining linear regression equations between *in vitro* digestibilities and porcine ileal apparent digestibilities. Dialysis cells have been more recently used in the estimation of the protein digestibility of novel food protein sources, such as seaweeds, where the high fiber content affected protein digestibility, likely by reducing the accessibility of the proteolytic enzymes (76, 77).

In some works, the use of chromatography or ultrafiltration with different cut off membranes has been used to characterize the digestible fraction. Besides, the characterization of the non-dialyzed digest has been conducted by ion-exchange or size exclusion chromatography, and ultrafiltration. The undigested residues were separated by ion-exchange chromatography into basic-neutral, lightly acidic and acidic fractions further resolved by sequential ultrafiltration (cut-off 10 and 1 kDa). Interestingly, large proportions of leucine, lysine, arginine, phenylalanine and tyrosine were found as part of peptides smaller than 1 kDa, both in the dialysates and retentates, while glutamine, threonine, serine and asparagine appeared mostly in fractions >1 kDa, while after 6 h with pancreatin, most of the proline appeared in the basic-neutral fraction >1 kDa (78). When this procedure was applied in the comparison of casein, cod, soy and gluten proteins, animal proteins were digested at a greater rate than plant proteins, and more resistant peptides were largely rich in proline and glutamic acid (79).

The impact of cooking on animal and plant protein digestion has been evidenced by the use of this strategy. The increase in protein digestibility of white and brown beans (*Phaseolus vulgaris*) after cooking was found to be related to a higher extent of proteolysis, as monitored by SDS-PAGE and recovery of low molecular weight peptides (< 30 kDa) after ultrafiltration of the digests (80). On the contrary, meat protein digestion in a microreactor fitted with a 10 kDa cut-off membrane in the gastric compartment and 1 kDa cut-off dialysis membrane in the intestinal compartment showed a decrease of protein digestibility with meat cooking (81). This study showed superior precision with the use of a semi-automatic flow procedure in comparison with the test tube method. Analytical size exclusion chromatography has been used to determine digestibility of casein *vs* modified casein with the glycation product pyrraline. The size pattern was used to show that the digestibility decreased with increasing pyrraline concentration of the peptide mixtures. Moreover, further ultrafiltration of digests using 1 kDa cut-off indicated that 50–60% of pyrraline was included in peptides (82).

The methods based on *in vitro* digestion and dialysis or ultrafiltration have shown good correlation in the prediction of ileal protein digestibility of food and feed. Some of these approaches have been used to characterize the gastrointestinal digests in combination with chromatographic methods. Main weaknesses of these methods would derive from the limited reproducibility of the use of ultrafiltration devices and the unspecific bound of protein material to the membrane material.

### 3.4 Dynamic systems

Dynamic systems have been proposed as *in vitro* alternatives for human or animal studies as physiologically relevant, including peristaltic mixing of food, computer- controlled pH values and realistic gastrointestinal transit times. Moreover, small molecules are removed from the digesta with hollow fiber membranes. The TNO-developed TIM^®^ system was tested to predict the true ileal digestibility of proteins including dairy, meat, wheat, faba bean or barley, and a linear relationship versus pig or calf data was obtained (83). A standard corn-based diet was compared with the same diet with coarse ground corn, 8% sugar beet pulp, 10% wheat bran, or 8% sugar beet pulp and 10% wheat bran. The dynamic model yielded digestibility coefficients comparable with *in vivo* ileal digestibility in growing pigs for the standard and coarse ground corn but the values were considerably affected by the incorporation of the fibrous ingredients. The linear fitting between the *in vitro* and the *in vivo* results for crude protein digestibility was not significant but resulted in *R*^2^ = 0.99 when the coarse ground corn diet was excluded from the regression (63).

Using the tiny-TIM, digestibilities of ovalbumin, cooked and raw chicken egg white, and casein showed similar values to values reported in humans (*R*^2^ = 0.96). The true ileal protein and amino acid digestibilities were used by the authors to estimate the DIAAS for immature herring egg proteins (84). More recently, cumulative true ileal digestibility of nitrogen data has been reported during 5 h tiny-TIM, expressed as the percentage of the exogenous nitrogen intake, correcting for nitrogen in gastric residue (85). These values served to calculate DIAAS for different protein ingredients, alongside the corresponding limiting amino acid. The DIAAS values for rice, whey, and pea-based proteins were in agreement with those collected from literature, using pig ileal data. However, for soy and a second source of pea protein with different processing, the values were significantly lower than those previously described in literature. This was ascribed to treatments applied to these specific ingredients during processing, including alkaline or heat treatment, leading to protein aggregation or structural changes. An alternative source of discrepancy was related to differences in the innate protein features due to cultivar of growing conditions. A low (under 50%) bioavailability of the majority of amino acids and low N digestibility was found for the last two products. Isolates with lower DIAAS also showed lower protein solubility and increased protein aggregation, which was identified as a potential cause inhibiting digestion. Indeed, DIAAS positively correlated to protein solubility and N-bioaccessibility. The dynamic system developed at INRAE, DIDGI^®^ was set up to mimic infant digestion upon an extensive analysis of literature on infant physiology and validated with piglet digestion (86). This system provided comparable results *in vitro/in vivo* for a reference dairy infant formula in terms of limiting essential amino acid, PDCAAS-like score and *in vitro* apparent digestibility. The last parameter was determined based on the soluble N lower than 10 kDa, as measured in the peptides by size exclusion chromatography and cumulated to the free amino acid nitrogen (64).

The use of dynamic systems to determine protein digestibility is still limited. Although these systems allow monitoring the progress and digestion kinetics, the calculation of the nitrogen mass balance could be more complex than in static systems. In addition to the difficulties to harmonize conditions in different apparatus, the availability of this sophisticated equipment could be an additional limitation to the extensive use of these methods.

## 4 INFOGEST static protocol applied to protein digestibility and protein quality analysis (*in vitro* DIAAS)

**Figure 1:**
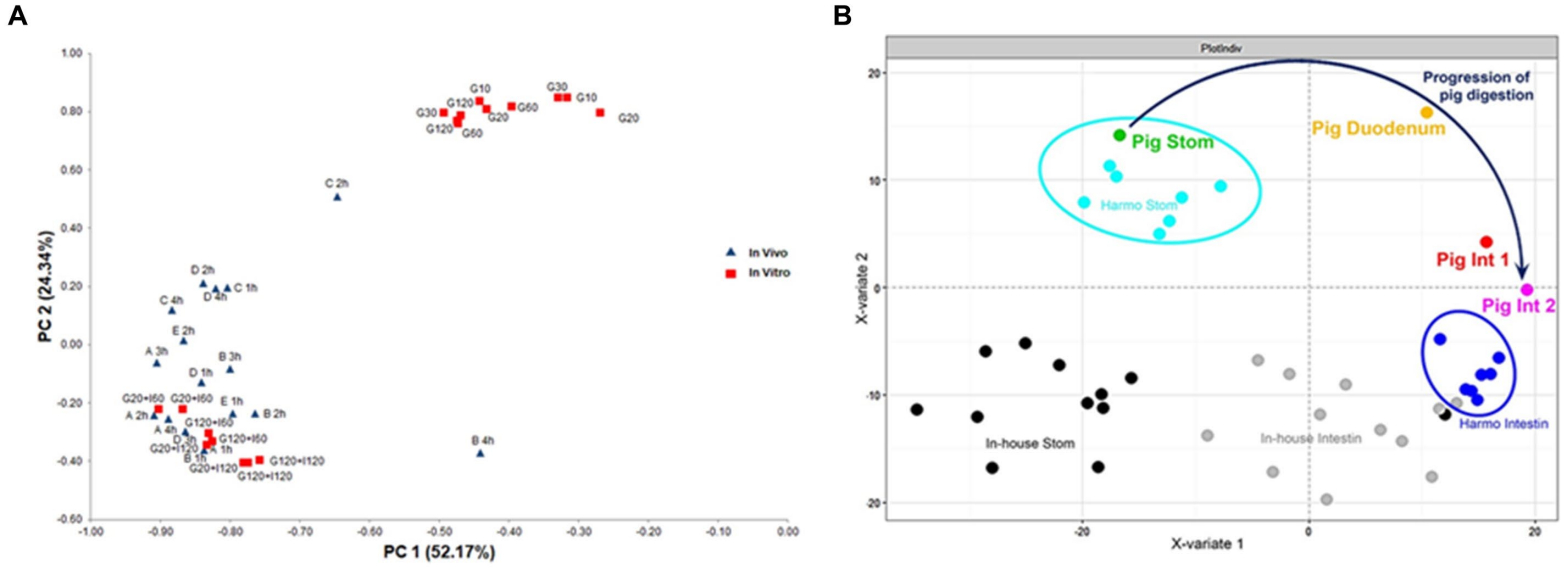
Comparison of *in vitro* digestion (INFOGEST protocol) *vs in vivo* (**A**: human jejunal digests; **B**: pig digests). **(A)** Principal component analysis score plot calculated with the frequency of appearance of each amino acid identified as part of a peptide from β-casein and α_s1_-casein. Different human subjects (blue triangles) are referred to with capital letters from A to E followed by the time of jejunal sampling (1, 2, 3, and 4 h). *In vitro* digests are represented with red squares. G, gastric; I, intestinal, followed by the time expressed in minutes of *in vitro* digestion. Reprinted with permission from Elsevier, by Sanchón et al. (5); **(B)** Partial least square analysis over all peptide patterns identified in the five most abundant milk proteins (β-, α_s1_-, α_s2_-, κ-casein, β-lactoglobulin). The average of eight pig samples is shown versus the harmonized or in-house digestion protocol, from previous interlaboratory studies. The arrow indicates the progression of digestion in the pig samples from Stom (stomach)-, Duodenum, Int 1 (proximal jejunum)-, to Int. 2 (median jejunum)- phases **(B)**. Reprinted with permission from Taylor & Francis, by Bohn et al. (17).

The INFOGEST static digestion protocol was developed during the COST Action INFOGEST^1^ with the main goal to harmonize the highly variable protocols used within the research laboratories interested in food digestion. The first INFOGEST consensus method (32), was followed by an improved and more detailed protocol in 2019 (33). Digestion parameters were based on currently available physiological data. The resultant peptides from the *in vitro* digestion with the INFOGEST protocol have been compared with human and pig peptidomic analysis showing comparable results for milk proteins (Figure 1). Compared to previous published protocols, the following points can be highlighted as the most important advantages, which helped to reduce experimental variability and improve reproducibility (87). Firstly, the protocol includes specific enzyme activity assays in order to harmonize the addition of enzymes based on their activity and not based on weight, as in previous published protocols. Secondly, due to the variable buffering capacity of different foods, the protocol requests to perform a pH test tube where the volumes of HCl to add in the gastric phase (to reach pH 3) and the volume of NaOH (to reach pH 7) in the intestinal phase, are tested for each food sample. And thirdly, the protocol provides indications on how the enzyme activities can be stopped after the gastric and intestinal phase of digestion, depending on the downstream analyses. The protocol was shown to be reproducible and robust in inter-laboratory experiments (87) and the results at the end of the intestinal phase were comparable to *in vivo* results (5, 88) although this has been proved so far only for dairy proteins.

Although the INFOGEST protocol increased harmonization of digestion experiments, critical steps in the protocol and further adaptations were proposed. The INFOGEST sub-group (WG4) tested lipase activity in several collaborative studies and found a high variability due to unprecise descriptions in the original protocol. The detailed protocol elaborated by this group led to a significant reduction in variability and for the study of lipid digestion, therefore these recommendations should be considered (89). A similar work focusing on amylase activity is currently ongoing and in the near future an improved protocol for amylase activity will be proposed and published. Moreover, in order to better simulate digestion in different age groups, both protocols, the static and the semi-dynamic protocol were and are further adapted to infant and elderly conditions.

The static INFOGEST *in vitro* digestion protocol represents a good starting point on which the quantification of the protein digestibility could be based. Several recent publications based on the INFOGEST static protocol are focusing on the quantification of protein digestibility and are listed chronologically in Table 2. In this table, the main adaptations with regard to the original INFOGEST method, the amount of protein input, the separation of non-digestible from digestible material, the use of an enzyme blank, and the calculations of digestibility are compiled. The approaches to overcome the main challenges of the *in vitro* methods are discussed below.

**Table 2:** *In vitro* methods based on the INFOGEST digestion protocol applied to the evaluation of protein and amino acid digestibility.

Food substrate	INFOGEST protocol, adaptations	Protein input	Separation of undigestible from digestible part	Enzyme blank	Calculation of digestibility	Comparability with *in vivo* data	Reference
9 protein concentrates: blood, corn, mealworm, Mycoprotein^®^, yellow peas, potato, whey, yeast	pH adjusted continuously by stat titration; 10 U/mL trypsin activity; sodium chloride instead of sodium bicarbonate	5%	Centrifugation + Ultrafiltration 5 kDa	H_2_O	Three different calculation strategies using total AA in the filtrate	No direct comparison	(90)
6 food products: cooked beef, raw chicken, wheat flour bread, heated/non-heated pea concentrate, casein	None	17%	Centrifugation + precipitation with TCA 8.3%	H_2_O	Small peptides determined by SEC area relative to the total protein	No direct comparison	(91)
7 food products: whey protein isolate, zein, collagen, black beans, pigeon peas, All-Bran^®^, peanuts	Supernatant of pancreatin suspension after ultrasound and centrifugation	4%	Precipitation with 80% methanol	Protein-free substrate containing fat, carbohydrates, and cellulose	Three analytical workflows: Total N or total AA or primary amines in the absorbable fraction relative to total digest corrected for protein-free substrate blank	Comparison for 7 same substrates with *in vivo* data: Digestibility, average difference: 1.2%, DIAAS, average difference: 0.1%	(92)
12 food products: 6 milk protein products, pea, soy, wheat, zein, cricket, mealworm	none	16%	TCA precipitation (6, 9, 12, and 15% + extraction of supernatant with diethyl ether)	Simulated fluids	N content in digestible vs. whole digesta corrected for N content of the blank and NPN content of the protein material	No direct comparison between foods, correlation of 0.912 for 12% TCA (linear regression)	(93)
Gluten and casein at 4, 8, 16% of the model meal	No oral phase	4, 8, 16%	Centrifugation + Ultrafiltration 10 kDa	Use of ^15^N labeled substrates	Total N in (<10 kDa) permeate relative to total N in food corrected for blank (<10 kDa) permeate	*In vitro* values below reported *in vivo* values	(94)
5 protein matrices: faba bean, pea flour, soy flour, whey protein isolate, casein	Addition of jejunal-ileal digestion phase, mimicking the brush border digestion: 13 mU/mL leucyl aminopeptidase, pH 7.2, 37°C, 4 h	Dependent on substrate	Centrifugation	H_2_O	Total AA in digest relative to total AA in food corrected for total AA in blank	*In vitro* underestimates *in vivo* values	(95)

AA, amino acids; DIAAS, digestible indispensable amino acid score; N, nitrogen; SEC, Size exclusion chromatography; TCA, tricholoacetic acid.

### 4.1 Enzyme/substrate ratio

The original INFOGEST protocol proposed for each digestion step a 1:1 ratio (w:w) between food and simulated fluid, ending up with a final ratio of 1:8 of food in digesta. No recommendation of nutrient normalization was proposed. However, in order to compare protein digestibility of different foods, a normalization may be needed. In four of the listed publications, protein input was normalized between 4 and 16% in the foods subjected to digestion. Increasing the amount of protein entering into the system reduced digestibility, as was observed in the case of different amounts of TCA soluble casein after size exclusion chromatography (91) and for casein and gluten digestibility, testing 4, 8, and 16% of protein input (94). Another approach to increase the food to enzyme ratio is the adaptation of digestive enzymes as proposed by Ariëns et al. (90) to reduce the background of enzymes. The authors reduced the addition of pancreatin from 100 U/mL of digesta by a factor of 10 to 10 U/mL and observed no impact on released NH_2_ during digestion of whey protein isolate, which represents a highly digestible substrate. It would be interesting to test if this observation is also correct for substrates with lower digestibility. Alternatively, a reduction in enzyme background was achieved by Sousa et al., by using the supernatant of the pancreatin suspension after solubilization with ultrasound and subsequent centrifugation (92). This procedure did not reduce the trypsin activity in the pancreatin supernatant.

### 4.2 Separation of digestible from non-digestible material

At the end of the intestinal phase of the original INFOGEST protocol, all products are in the same container. In order to assess protein digestibility, digestible and non-digestible fractions need to be separated. Different approaches were used by various authors, such as centrifugation (95), or ultrafiltration at different cut-off sizes, such as 5 or 10 kDa (90, 94), corresponding to peptides of 45–90 amino acids in length, assuming an average weight of 110 Da per amino acid. The choice of the rather high molecular weight cut-off compared to *in vivo* (500 Da) was justified by the lack of brush border enzymes in the system (94). Moreover, the use of ultrafiltration could as well lead to loss of material, impacting the mass-balance and in consequence the digestibility of the tested substrates (90). A second approach applied in the different protocols was a precipitation step either with different concentrations of TCA (6–12%) (91, 93) or with MeOH (80%) (92). Depending on the downstream analysis, the precipitation agent could disturb the measurements and it was removed by extraction with diethyl ether (93) or simply be evaporated in the case of MeOH (92).

### 4.3 Consideration of the enzyme background

In both, *in vivo* and *in vitro* situations, the endogenous enzymes and background proteins need to be considered. Different solutions to this challenge have been suggested. Probably the most precise way of differentiating endogenous material from the food of interest represents the use of isotopically labeled food sources as has been used by Ménard et al. (94). In this study, ^15^N isotopically labeled casein and gluten were digested at different concentrations. Unfortunately, the generation of isotopically labeled substrates is not always possible, in addition to being time consuming and expensive, and therefore other solutions are requested. However, for experiments of proof of principle and validation, isotopic labeling would be the method of choice. As an alternative, the enzyme background was subtracted by performing a parallel digestion with H_2_O (90, 93–95) or using a protein-free food (92). However, as explained in Section 2, in the absence of substrate, a higher enzyme autolysis may occur (39, 94), which would cause an underestimated digestibility value.

### 4.4 Validation and standardization of *in vitro* protein digestibility protocols

**Figure 2:**
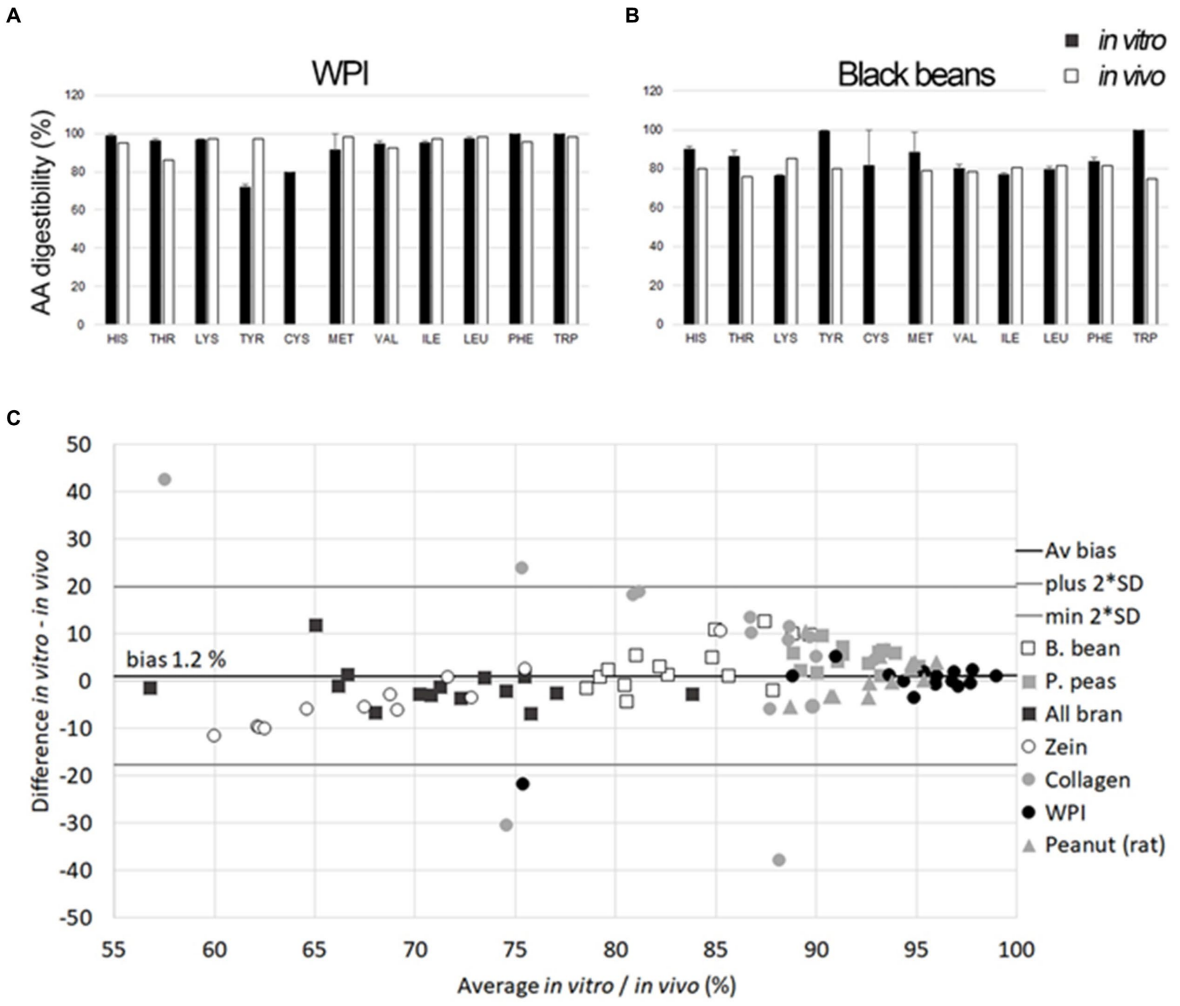
*In vitro* digestibility (y-axis: %) of individual amino acids (black) after IVD compared with *in vivo* data for whey protein isolate (WPI) **(A)** and B. bean **(B)** (mean pig and human values, white). Error bars are SEM of three individual *in vitro* experiments; Statistical comparison between *in vitro* and *in vivo* digestibility of essential amino acids AA **(C)**, according to previous work (96), show the average digestibility of *in vitro* and *in vivo* results (x-axis) versus the differences between *in vitro* and *in vivo* digestibility (y-axis) of all essential amino acids of the comparisons of B. bean, P. peas, All bran, Zein, Collagen, WPI, and Peanut [*in vivo* rat data, (97)]. The mean bias between methods was 1.2% and upper and lower limits indicate ±2 * SD of the average difference. The comparison with *in vivo* DIAAR for SAA could not be calculated due to missing *in vivo* cysteine values.

Comparisons between *in vitro* and *in vivo* data were performed by four of the above-mentioned publications (Table 2). A high correlation between *in vitro* and *in vivo* DIAAS, as well as an agreement in limiting amino acid (DIAA) was demonstrated for the investigated substrates, although the *in vitro* digestibility values were below *in vivo* digestibilities in these studies (93, 95). In the same direction, a lower true *in vitro* digestibility value compared to *in vivo* was found for the two investigated substrates, casein and gluten (94). A direct comparison between *in vivo* and *in vitro* digestibility was performed for seven substrates (Figure 2, WPI (A) and black bean (B), representing two of the investigated substrates). The results showed a comparable digestibility with an average bias of 1.2% for all essential amino acids of the assayed substrates (Figure 2C). The DIAAS values were comparable with an average bias of 0.1% and a correlation of r = 0.96 between *in vivo* and *in vitro* results (92). It has to be highlighted that this latter study was carried out *in vivo* and *in vitro* by using identical substrates. In view of the published data, an increased number of substrates of different nature is needed to validate this *in vitro* model for digestibility.

### 4.5 Method repeatability, reproducibility, and standardization

Until recently, a major drawback of *in vitro* methods was the lack of comparability between different laboratories. In consequence, one of the major achievements of the INFOGEST network was to establish harmonized digestion protocols with satisfactory inter-laboratory reproducibility. Within the same INFOGEST network, protein digestibility is currently tested with several dairy products (SMP, whole milk powder, whey protein isolate, yogurt, and gruyere cheese) and with two plant sources (soy protein isolate, chickpea), applying the analytical workflow published by Sousa et al. (92). In parallel to these collaborative studies, a standardization of the method within the International Dairy Federation (IDF) and International Standardization Organization (ISO) was launched. The precision data (repeatability, reproducibility) obtained in the inter-laboratory trials will be included in the future IDF/ISO standard method, with the final goal to obtain a robust and validated protocol allowing the analysis of protein digestibility.

## 5 Conclusions and future prospects

Several *in vitro* methods to be applied in the assessment of the protein nutritional quality have been developed during the last 40 years. *In vitro* digestion models have been shown to provide a good estimation of protein digestibility and of the nutritional scores, such as the DIAAS, and appear to be a realistic alternative to animal trials in the near future. Some of them have demonstrated good agreement with *in vivo* digestibility data with high correlation coefficients or close protein and amino acid digestibility values. It is important to note that most of these correlations were established protein fecal protein digestibility, while these methods do not take into account the action of microbiota, and thus, when possible, the comparison with standardized or true ileal digestibility data is preferred.

Despite the huge effort done, *in vitro* methods have not reached sufficient confidence to be used for the routine evaluation of protein and amino acid digestibility due to discrepancies in certain substrates. The conditions of static *in vitro* methods are fixed, in the most optimal situation by mimicking as closely as possible the digestive conditions: enzyme/substrate ratios, standardized enzymatic activity, and a digestion time, etc. However, it is highly unlikely that the *in vitro* conditions will be able to simulate all types of foods, matrices, and ingredients without adaptations. For instance, the work performed to date with the INFOGEST method has already detected the need to test protein isolates the same as done *in vivo*, i.e., incorporated in a protein-free food matrix. Similarly, substrates with low protein content or having a high content of trypsin inhibitors will require protocol adaptations. Therefore, it is crucial to carry out *in vitro* protein and amino acid digestibilities of a wide range of substrates with previously measured ileal digestibilities in order to identify limitations and propose adaptations to the *in vitro* protocols. In this sense, new *in vivo* data obtained on biological fluids are needed to refine these *in vitro* digestion conditions. Such work is currently being completed in the frame of a cooperation between INFOGEST and the UNGAP network on drug absorption. Moreover, a large proportion of the studies comparing *in vitro* to *in vivo* values has been made on protein ingredients from animal, plant or alternative sources, although humans do not consume ingredients, but food that adds complexity. More work is needed to apply *in vitro* models to determine protein digestibility on real food where the other constituents of the food matrix can interact with each other, especially when they are processed or ultra-processed, and can limit the accessibility of digestive enzymes to their protein substrates.

Static *in vitro* digestion models are relatively simple techniques with a huge potential for assessing protein digestibility. However, based on the experience within the INFOGEST network, even with protocols extensively described step by step, some slight differences may lead to significant discrepancies. It is important that the validation of these *in vitro* methods is run in different laboratories to generate enough reproducibility and repeatability data. A huge effort is being done in INFOGEST to train people on how to use the model in a proper way and training schools organized in Europe, South America, Australia and Canada. Videos showing the different steps of the model, the digestive enzyme calibration or the quantification of bile salts have been made available on the INFOGEST YouTube channel.^2^ All these events and tools will highly improve the reproducibility of the model, leading to more robust interlaboratory data.

## Author contributions

GS-S: Writing – original draft, Writing – review & editing, Investigation, Formal analysis. BM: Writing – original draft, Writing – review & editing, Resources. AB: Writing – original draft, Writing – review & editing. DD: Writing – original draft, Writing – review & editing. LE: Writing – original draft, Writing – review & editing. IR: Writing – original draft, Writing – review & editing, Conceptualization, Funding acquisition, Supervision, Project administration.

## Footnotes

^1^ http://www.cost-INFOGEST.eu/

^2^ https://www.youtube.com/@fooddigestion
